# Wellbeing while waiting evaluating social prescribing in CAMHS: study protocol for a hybrid type II implementation-effectiveness study

**DOI:** 10.1186/s12888-023-04758-0

**Published:** 2023-05-10

**Authors:** Daisy Fancourt, Alexandra Burton, Feifei Bu, Jessica Deighton, Richard Turner, Joely Wright, Alexandra Bradbury, Marc Tibber, Shivangi Talwar, Daniel Hayes

**Affiliations:** 1 Research Department of Behavioural Science and Health, Institute of Epidemiology & Health Care, Social Biobehavioural Research Group, University College London, 1-19 Torrington Place, London, WC1E 7HB UK; 2 Evidence Based Practice Unit, University College London and the Anna Freud National Centre for Children and Families, 4-8 Rodney St, London, N1 9JH UK; 3 Department of Clinical, Educational and Health Psychology, Division of Psychology and Language Sciences, 1-19 Torrington Place, London, WC1E 7HB UK; 4 Division of Psychiatry, University College London, Maple House, Gower Street, London, WC1E 6BT UK

**Keywords:** Social prescribing, Wellbeing, Link worker, Mental Health, CAMHS, Child, Young person

## Abstract

**Background:**

Social prescribing is a mechanism of connecting patients with non-medical forms of support within the community and has been shown to improve mental health and wellbeing in adult populations. In the last few years, it has been used in child and youth settings with promising results. Currently, pathways are being developed for social prescribing in Child and Adolescent Mental Health Services (CAMHS) to support children and young people on treatment waiting lists. The Wellbeing While Waiting study will evaluate whether social prescribing benefits the mental health and wellbeing of children and young people.

**Methods:**

This study utilises an observational, hybrid type II implementation-effectiveness design. Up to ten CAMHS who are developing social prescribing pathways as part of a programme run across England with support from the Social Prescribing Youth Network will participate. Outcomes for children and young people receiving social prescribing whilst on CAMHS waiting lists will be compared to a control group recruited prior to the pathway roll-out. Questionnaire data will be collected at baseline, 3 months and 6 months. Primary outcomes for children and young people are mental health symptoms (including anxiety, depression, stress, emotional and behavioural difficulties). Secondary outcomes include: loneliness, resilience, happiness, whether life is worthwhile, life satisfaction, and service use. An implementation strand using questionnaires and interviews will explore the acceptability, feasibility, and suitability of the pathway, potential mechanisms of action and their moderating effects on the outcomes of interest, as well as the perceived impact of social prescribing. Questionnaire data will be analysed mainly using difference-in-differences or controlled interrupted time series analysis. Interview data will be analysed using reflexive thematic analysis.

**Discussion:**

The Wellbeing While Waiting study will provide the first rigorous evidence of the impact of social prescribing for children and young people on waiting lists for mental health treatment. Findings will help inform the prioritisation, commissioning, and running of social prescribing in other CAMHS. To maximise impact, findings will be available on the study website (https://sbbresearch.org) and disseminated via national and international networks.

**Trial Registration:**

N/A.

## Introduction

Children and young people’s (CYP’s) mental health is one of the greatest challenges facing the United Kingdom (UK) National Health Service (NHS). Prior to the COVID-19 pandemic, it was estimated that 1 in 9 young people had a diagnosable mental health condition [1]. Since COVID-19, that figure has increased to 1 in 6, with 80% of young people saying the pandemic has made their mental health worse [2]. Yet less than 1% of the total NHS budget is spent on Child and Adolescent Mental Health Services (CAMHS) [1]. Additionally, it is widely recognised that government plans to prevent, mitigate and respond to the rise in mental health demand as a result of the pandemic were insufficient [3, 4]. Thus, there is a clear need for additional support to meet the country’s youth mental health needs.

Social prescribing is a mechanism of care used since the 1980s in the UK and was formally launched as a national programme by NHS England in 2018 to link patients with non-medical forms of support within the community [5]. This process usually involves a health or social care professional referring a patient to a Social Prescriber, sometimes known as a Link Worker (LW), who develops a non-clinical plan that connects the patient with community organisations to improve health, wellbeing or other aspects of the patient’s life [5]. Activities include the arts, cultural events, and other support services, such as physical activity, financial support, volunteering and befriending. Social prescribing has the potential to play an important role as an alternative or adjunct to conventional medical interventions for mental health by:

i integrating psychological, social and biomedical approaches to improving mental health, so can provide more person-centred care and help address determinants of mental illness [6];
ii offering an alternative to pharmacological prescribing, which is especially pertinent for CYP given ongoing debates about appropriateness of many psychiatry medications for this age group [7, 8];
iii providing support from broader services, helping to reduce pressure on stretched mental health services [9];
iv aligning with shifts in thinking about ‘recovery’ in mental illness, moving from the notion of ‘cure’ or symptom remission[10, 11] to finding and maintaining hope, agency, independence, purpose, and integration with peers and communities [12];
v focusing on community engagement (avoiding languages of pathology), which can help mental illness seem less ‘abnormal’, decrease stigma and shame, and reduce barriers to seeking help [12];
vi Supporting the ethos of personalised care by giving CYP more control and choice in their mental health support [6].

NHS England announced in 2018 that it would be rolling out social prescribing as a component of Universal Personalised Care, with nearly 1 million people receiving referrals by 2023/24 [13]. Since the 2018/19 financial year, there has been a greater than 12-fold increase in the rate at which social prescribing for mental health was used in England [14]. However, despite the NHS Long Term Plan stating that social prescribing would be an “all-age model, from maternity and childhood through to end of life”, CYP have largely not been engaging in social prescribing [15]. There are a number of possible reasons for this. For example, CYP and their parents/guardians may not know about or understand social prescribing, or LWs may not feel confident working with CYP and connecting them with appropriate community activities in their areas [16]. Additionally, much of the emphasis on the roll-out of social prescribing through NHS England has focused on the General Practitioner-Link Worker (GP-LW) model, which may not be the best way to administer social prescribing to this population due to CYP’s perceptions of whether GPs can help with their mental health [17].

There is also a further challenge in CYP social prescribing: the evidence base on efficacy is in its infancy. A review from 2020 of scientific papers and grey literature found no studies on social prescribing in CYP populations [18]. Since then, an updated review [19] has identified a growing evidence base that indicates that social prescribing could both help improve mental health and wellbeing and have a favourable return on investment given that the costs of social prescribing schemes are lower than the relative value of its outcomes, including mental health, education, employment and volunteering. However, the review recommended that further robust research, including utilising control groups and larger samples, was needed, as well as further work with younger adolescents where the evidence base was less developed.

Whilst benefits are now being widely reported amongst adults such as improved wellbeing and quality of life, reduced social isolation and loneliness, and reduction in health service utilisation/costs [20–25], the different “social ecology” of CYP mental health means that extrapolation from adult studies to CYP cannot be made simply [26]. Further, social prescribing remains challenging to evaluate because it is not a single intervention but a mechanism of care [27]. Nonetheless, there is reason to believe that social prescribing could have benefits for CYP. First, benefits from many of the activities involved in social prescribing (e.g. music, dance, and other arts and cultural events) have been widely reported by observational and experimental studies [28]. Second, there is a strong theoretical rationale for why the arts, leisure and culture could support mental health, with over 600 mechanisms of action identified to date, including psychological, biological, social and behavioural pathways [29]. Third, CYP have shown broadly positive attitudes to social prescribing in various studies, especially as it provides a new social sphere for engagement, aiding in self-transformation (e.g. building skills and agency) and providing holistic alternatives to medical models of care [30].

In light of these potential benefits, there are increasing numbers of CAMHS sites that are integrating social prescribing into care pathways and offering, for example, social prescribing to CYP on waiting lists for treatment. However, it is vital to ascertain whether such social prescribing programmes have benefits for CYP, their parents/guardians and for CAMHS. The Wellbeing While Waiting study seeks to identify whether this is the case using an observational, hybrid type II implementation-effectiveness design. Specifically, the study will observe CAMHS sites that are working with the Social Prescribing Youth Network (SPYN)’s social prescribing programme, which is being piloted at selected CAMHS sites across England. The findings could support the work of dozens of CAMHS nationally that are piloting similar schemes or looking to replicate the SPYN programme.

## Methods

Drawing on an observational, hybrid type II implementation-effectiveness design, the Wellbeing While Waiting study seeks to identify if CYP on CAMHS waiting lists benefit from social prescribing programmes, and explore factors which affect implementation of the pathway, using questionnaires and interviews.

### Primary objective

The primary objective is to explore whether social prescribing pathways in CAMHS impact the mental health of CYP.

### Secondary objectives

The secondary objectives are to explore whether social prescribing pathways in CAMHS:

i impact the wellbeing and social experiences of CYP.
ii impact service-level outcomes.
iii support the mental health, wellbeing and social experiences of parents/guardians.
iv are acceptable, suitable, and feasible.
v have anticipated uptake, fidelity, and potential for long-term success.
vi have anticipated costs in delivery and have any barriers to access.
vii require specific implementation factors to achieve positive benefits.

### Design

Wellbeing While Waiting is a cohort observational study with both control and intervention arms. To answer the research objectives, a mixed methods approach will be undertaken using quantitative (questionnaire) and qualitative (interview) approaches.

### Setting of the study

Up to 10 CAMHS sites in England that are launching a pathway in 2023 with the SPYN’s social prescribing programme will be selected to participate in the study. Sites will be purposefully selected for variation based on geographical location, waiting list time, if they already participate in social prescribing, and if they are part of the i-THRIVE [31] transformation programme in CAMHS.

### Participants

Study participants include 600 CYP aged between 11 and 18 who are on waiting lists at CAMHS, up to 40 parents/guardians, and 80 LWs/Social Prescribers and CAMHS clinicians.

### Eligibility criteria

#### Participating sites

To be eligible, CAMHS must have a SPYN social prescribing pathway due to launch in 2023, be located in England and agree to participate in the study. Sites will be excluded if they do not have a SPYN social prescribing pathway due to launch in 2023.

#### CYP

CYP participants must be aged between 11 and 18 years old, have the capacity to give assent (if under 16 years old) or informed consent (if aged 16 or over), and have been on the CAMHS waiting list for less than one month. CYP’s treatment will not be altered or postponed in any way due to them taking part in social prescribing. CYP with eating disorders, psychosis or severe and complex difficulties (judged by the assessing clinician) will be excluded from participation in the study.

#### Parents/guardians

Parent/guardian participants must be aged 18 or over, have the capacity to give informed consent for their own participation in the study, and have capacity to give informed consent for CYP involvement in the study (when the CYP is aged between 11 and 15 years old).

#### LWs/social prescribers and CAMHS staff

LWs/Social Prescribers and CAMHS staff must be aged 18 or over, be working in or with one of the recruited sites and have the capacity to give informed consent to participate in the study.

### Study measures

#### Children and young people

Demographic information, socio-contextual information, and perceived mental health difficulties.

Primary outcome measures

- Revised Children’s Anxiety and Depression Scale (RCADS) [32].
- The Perceived Stress Scale [34].
- Strengths and Difficulties Questionnaire (SDQ) and Impact (self-report) [33], which measures emotional and behavioural difficulties.

Secondary outcome measures

- An item on whether they feel lonely (self-created).
- Subscales from the Student Resilience Survey (SRS) [35].
  - Community connection.
  - Participation in community life.
  - Self-esteem.
  - Empathy.
  - Problem solving.
  - Goals and aspirations.
- Office for National Statistics (ONS) items on feeling happy, worthwhile and satisfied [36].
- Service use as measured by the Client Service Receipt of Inventory (CSRI) [37].
- Acceptability of intervention measure (AIM) [38].

#### Parents/guardians

Qualitative interviews will explore CYP and parent/guardian experiences of the CYP receiving social prescribing including how, if at all, social prescribing has led to any changes for the CYP as well as what is helpful for the CYP’s mental health and barriers/enablers for engagement. The topics for those in the control group will focus on experiences of waiting and any support received.

#### Link Workers/Social Prescribers

- Acceptability of intervention measure (AIM) [38].
- Intervention Appropriateness Measure (IAM) [38].
- Feasibility of Intervention measure (FIM) [38].
- Perceived risks and strengths of factors known to impact long term success of service initiatives - The Long Term Success Tool (LTST) [39].
- Session Feedback Questionnaires.
- Goal based outcomes.
- Number of sessions they worked with the CYP and activities the CYP engaged in.

#### CAMHS staff

- CYP mental health diagnosis.
- Retention of CYP on the CAMHS pathway (whether a CYP stays on the waiting list vs. withdraws).
- Deterioration of mental health in CYP on CAMHS pathways (leading to CYP moving onto more intensive specialist pathways).
- Any mental health interventions received whilst on the CAMHS social prescribing pathway.
- The length, duration and impact of the psychological intervention when received.

Qualitative interviews with link workers/social prescribers and CAMHS staff will explore their perceptions of the pathway and how, if at all, social prescribing has led to any changes for the CYP, any barriers/facilitators to engaging in social prescribing, and factors around the embedding and sustainability of the social prescribing pathway.

### Procedure

#### Children and young people

In total, 600 CYP will be recruited to the study. This will consist of a control group of 200 CYP who will be recruited prior to the social prescribing pathway being finalised and rolled out at sites, and an intervention group of 400 CYP recruited via the social prescribing pathways.

Before social prescribing programmes are offered at each site, CAMHS staff will tell all new eligible CYP on waiting lists (and their parents/guardians where applicable) about Wellbeing While Waiting. Once the social prescribing programme is live, this will be amended so that CAMHS staff only tell CYP who accept a referral to the social prescribing pathway about Wellbeing While Waiting. Link Workers/Social Prescribers (for those who receive social prescribing) may also tell participants referred to them from participating CAMHS sites about Wellbeing While Waiting during a preliminary conversation with the CYP, if this has not been undertaken by CAMHS.

All CYP, including those who do and do not receive social prescribing, who consent to the study will be asked to complete questionnaires (see study measures) at baseline (i.e. when a CYP is first consented to the study), and 3 and 6 months later. Demographic information, socio-contextual information, and perceived mental health difficulties will be collected at baseline only. Questionnaires are self-administered and can be completed online, over the phone, or via videocall. In cases where none of these are feasible, the questionnaires will be completed in person with a researcher. Participants will receive a £10 voucher for completion of the questionnaire at each timepoint.

When they join the study, participants will be asked if they’d be happy to take part in an optional qualitative interview. From those who agree, up to 70 CYP (30 controls and 40 referred to social prescribing) will be selected using purposive sampling, taking into account socio-demographic factors, such as age, gender, ethnicity, site, mental health diagnosis (and for those receiving social prescribing, how much of the pathway they completed: none, some, still continuing). The interviews will predominantly take place over Microsoft Teams, but when this is not feasible for participants, telephone or in-person interviews will be offered.

The research team will also, with consent from CYP (and where applicable parents/guardians) draw on routine data held by CAMHS and LW/Social Prescriber host organisations. This will occur at 6 month follow up, once social prescribing has taken place. For Link Workers/Social Prescribers, data will include session feedback questionnaires, goal-based outcomes, the number of sessions had with CYP and the activities they engaged in. For CAMHS, this will include diagnosis, CYP mental health diagnosis, retention of CYP on the CAMHS pathway, any changes in clinical symptomology, any mental health interventions received, and the length, duration and impact of the psychological intervention when received.

#### Parents/guardians

The parents/guardians of CYP in the intervention group will be approached to take part in a qualitative interview. Up to 40 parents/guardians will be selected using purposive sampling, taking into account socio-contextual factors collected on the baseline questionnaire, such as parent/guardian educational attainment and members of the CYP household. The interviews will predominantly take place over Microsoft Teams, but when this is not feasible for participants, telephone or in-person interviews will be offered.

#### LWs/Social Prescribers and CAMHS clinicians

Once the pathway has been established (at least 6 months into the running of the social prescribing programme), up to 80 CAMHS clinicians and LWs/Social Prescribers will complete self-administered online questionnaires on the acceptability, appropriateness, and feasibility of social prescribing for CYP. Additionally, around 30 CAMHS clinicians and LWs/Social Prescribers will be invited to take part in a qualitative interview. Purposive sampling will be employed to select interviewees, taking into account socio-demographic factors, location, professional role, and types of activities offered by LWs/Social Prescribers. Interviews may be over the phone, in person, or via Microsoft Teams depending on staff preference.

For all questionnaires completed online, REDCap will be used. Audio recordings will, with consent from participants, be sent to an external transcription company approved by UCL. Any identifiable data will be stored in UCL’s Data Safe Haven which has been certified to the ISO27001 information security standard and conforms to NHS Digital’s Information Governance Toolkit.

### Sample size

In the absence of data to inform sample size calculations, the sample size has been selected based on the number of CYP anticipated to go through the social prescribing pathway in the amount of time we have to recruit within the research funding. A 1:2 control:intervention allocation has been selected as there is anticipated to be greater heterogeneity of experiences in the intervention group (i.e. through differences in attendance rates at social prescribing activities, as will be tracked through the implementation research measures).

For qualitative data collection the sample size has been selected to be large enough to allow for adequate “information power” and to make meaningful comparisons between social prescribing pathway delivery and experiences in different sites [40], but not too large to dilute an in-depth rich analysis and exploration of individual participant accounts.

### Analysis

#### Quantitative analysis

Quantitative data will be analysed using STATA 17 [41] or MPLUS 8 [42]. To test the impact of social prescribing on CYP and service outcomes, the research team will use difference-in-differences or controlled interrupted time series analysis as the primary analytical method. Subgroup analyses will also be explored (e.g., based on level of engagement in social prescribing and whether CAMHS treatment starts alongside social prescribing or not), accounting for baseline differences between these groups.

To test whether there is any evidence that specific demographic groups stand to benefit more from the social prescribing pathway, the research team will test moderation effects and conduct sub-group and sensitivity analyses.

To examine potential mechanisms of the implementation effectiveness, the research team will explore potential mediation and moderation effects using structural equation modelling or counterfactual approach to mediation/moderation analysis.

To assess representativeness, we will compare aggregate registration data for all CYP who are eligible for social prescribing with CYP who participate in social prescribing over the same time period (the research sample).

#### Qualitative analysis

Qualitative data will be managed using NVivo 12 [43]. The research team will conduct reflexive thematic analysis [44] to identify the potential mechanisms of action of social prescribing (how and why social prescribing may or may not impact mental health and wellbeing). For the controls, the analysis will explore experiences of waiting for CAMHS treatment and the different coping strategies employed whilst waiting. A core aim of the analysis of qualitative implementation data is to identify similarities and differences in implementation across the different CAMHS sites.

## Patient and public involvement

A Youth Advisory Group (YAG), comprising CYP who have lived experience of mental health problems and/or social prescribing has been convened. To ensure that expertise by experience informs all aspects of the study, the YAG have provided input into the choice of questionnaires, supported study design, and co-produced participant documentation, such as Participant Information Sheets. During the study recruitment phase, two paid summer internships will be offered to CYP who will provide input into data collection and analysis. The YAG will co-present the results at CAMHS and be invited to write blogs for the project website about their work. Where appropriate, we will involve the YAG in academic outputs, e.g., as co-authors on papers or as presenters of a YAG perspective on the study.

## Discussion

Wellbeing While Waiting has the potential to fill the evidence gap regarding social prescribing for CYPs mental health and wellbeing within CAMHS. There are a number of benefits to this. Primarily, it will provide the first rigorous evidence on the impact of social prescribing for child and youth mental health and wellbeing. Second, an understanding of the potential mechanisms of action will allow for the better implementation and efficacy of social prescribing in services and by LWs/Social Prescribers. Third, evidence of impact to mental health and wellbeing would show whether the roll-out of social prescribing across CAMHS could support a service which is currently overwhelmed. Lastly, evidence of benefit around social prescribing for children and young people with mental health problems could stimulate exploration of the potential of social prescribing for other conditions. Findings will be disseminated through conferences and journal articles, evidence briefings, blogs and vlogs for CYP, and via a festival at the end of the project.

## Data Availability

An anonymised quantitative dataset will be available in 2026 from the corresponding author on reasonable request. Full qualitative transcripts will not be publicly available due to containing information that might compromise the identity of research participants.

## Abbreviations

AIM: Acceptability of intervention measure
CAMHS: Child and Adolescent Mental Health Services
CYP: Children and Young People
CSRI: Client Service Receipt of Inventory
FIM: Feasibility of Intervention measure
IAM: Intervention Appropriateness Measure
LTST: The Long Term Success Tool
LW: Link Worker
NHS: National Health Service
ONS: Office for National Statistics
RCADS: Revised Children’s Anxiety and Depression Scale
SPYN: Social Prescribing Youth Network
SDQ: Strengths and Difficulties Questionnaire
SRS: Student Resilience Survey
UCL: University College London
UK: United Kingdom
YAG: Youth Advisory Group
